# How Darwinian is cultural evolution?

**DOI:** 10.1098/rstb.2013.0368

**Published:** 2014-05-19

**Authors:** Nicolas Claidière, Thomas C. Scott-Phillips, Dan Sperber

**Affiliations:** 1 CNRS, Fédération de recherche 3C, Laboratoire de psychologie cognitive, Université d'Aix – Marseille, 3 Place Victor Hugo, Bât. 9, Case D, 13331 Marseille cedex, France; 2 Evolutionary Anthropology Research Group, Department of Anthropology, Durham University, Dawson Building, South Road, Durham DH1 3LE, UK; 3 Department of Cognitive Science, Central European University, Nador u. 9, 1051 Budapest, Hungary; 4 Department of Philosophy, Central European University, Nador u. 9, 1051 Budapest, Hungary; 5 Institut Jean Nicod (CNRS, EHESS, ENS), 29 rue d'Ulm, 75005 Paris, France

**Keywords:** culture, cultural evolution, cultural attraction, population thinking

## Abstract

Darwin-inspired population thinking suggests approaching culture as a population of items of different types, whose relative frequencies may change over time. Three nested subtypes of populational models can be distinguished: evolutionary, selectional and replicative. Substantial progress has been made in the study of cultural evolution by modelling it within the selectional frame. This progress has involved idealizing away from phenomena that may be critical to an adequate understanding of culture and cultural evolution, particularly the constructive aspect of the mechanisms of cultural transmission. Taking these aspects into account, we describe cultural evolution in terms of *cultural attraction*, which is populational and evolutionary, but only selectional under certain circumstances. As such, in order to model cultural evolution, we must not simply adjust existing replicative or selectional models but we should rather *generalize* them, so that, just as replicator-based selection is one form that Darwinian selection can take, selection itself is one of several different forms that attraction can take. We present an elementary formalization of the idea of cultural attraction.

## 1. Population thinking applied to culture

In the past 50 years, there have been major advances in the study of cultural evolution inspired by ideas and models from evolutionary biology [1–8]. Modelling cultural evolution involves, as it would for any complex phenomenon, making simplifying assumptions; many factors have to be idealized away. Each particular idealization involves a distinct trade-off between gaining clarity and insight into hopefully major dimensions of the phenomenon and neglecting presumably less important dimensions. Should one look for the best possible idealization? There may not be one. Different sets of simplifying assumptions may each uniquely yield worthwhile insights. In this article, we briefly consider some of the simplifications that are made in current models of cultural evolution and then suggest how important dimensions of the phenomenon that have been idealized away might profitably be introduced in a novel approach that we see as complementary rather than as alternative to current approaches. All these approaches, including the one we are advocating, are Darwinian, but in different ways that are worth spelling out.

Much clarity has been gained by drawing on the analogy between cultural and biological evolution (an analogy suggested by Darwin himself: ‘The formation of different languages and of distinct species, and the proofs that both have been developed through a gradual process, are curiously parallel’ [9, p. 78–79]). This has made it possible to draw inspiration from formal methods in population genetics with appropriate adjustments and innovations. Of course, the analogy with biological evolution is not perfect. For example, variations in human cultural evolution are often intentionally produced in the pursuit of specific goals and hence are much less random than in the biological case. There are many such disanalogies between the way variation, selection and inheritance operate in the biological and cultural cases, and these have been readily acknowledged and taken into account in models of cultural evolution. The methodological advantage of staying close to biological models has, however, been a factor in deciding which specific features of cultural evolution to take into account and which to idealize away. The successes of this research do retrospectively justify the choices that were made, but at the same time they leave some deep questions unanswered.

We agree with Richerson & Boyd [8] that the overall general framework for the study and modelling of cultural evolution should be that of ‘population thinking’ (so named by Ernst Mayr, who described it as one of Darwin's most ‘fundamental revolutions in biological thinking’ [10]). Population thinking involves looking at a system (such as culture) as a population of relatively autonomous items of different types with the frequency of types changing over time. The types themselves are not defined by their ‘essence’ but as historical subpopulations, features of which may change over time.

**Figure 1.**
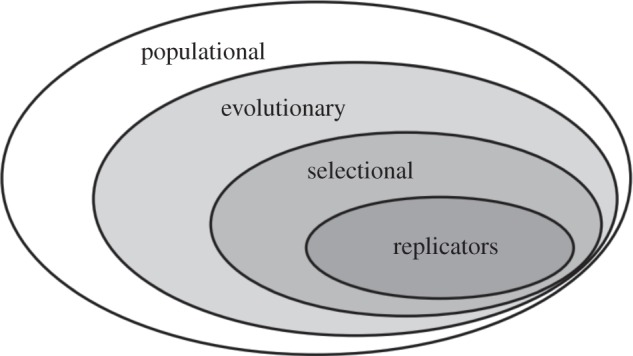
Four nested explanatory frameworks.

Within this wide Darwin-inspired populational framework, one may, adapting a suggestion and a diagram of Peter Godfrey-Smith's [11], distinguish three more specific nested explanatory frames (figure 1). First, a population is evolutionary if the frequencies of types of different items at any given time step are to a large extent explained as a function of their frequencies at earlier time steps. Second, a population that renews itself through the reproduction of its members is subjected to Darwinian selection if the items exhibit variation, heritability and fitness differences. Finally, within this selectional frame, a population is replicative if heritability is secured by some form of replication. This is Darwinian selection in its clearest form, clearer in fact than it was to Darwin himself. The label ‘Darwinian’ is often used in the restricted sense of the selection frame, but in fact all four frames are Darwinian, in different ways. Population thinking is ‘Darwinian’ in a broad sense and so is applying it to evolution; a focus on selection in explaining evolution is ‘Darwinian’ in a standard sense; and explaining heritability and its role in selection in terms of replicators is ‘Darwinian’ in a rationally reconstructed sense. For each of these frames, modelling is best done by ignoring or back-grounding processes outside of the frame.

Memetics argues that this last, replicative framework applies to cultural evolution as much as it does to biological evolution; memes are to culture what genes are to biology [12,13]. Replication however is not indispensable for selection, and the broader selectional frame, which includes cases of reproduction that fall short of replication, arguably provides richer explanations for the evolution of complex cultural phenomena and for the cumulative character of human culture [2,3,7,8]. So far, the modelling of cultural evolution has largely been developed within this selectional frame (but see e.g. [14] for an alternative approach and [15] for discussion of the limitations of the selectionist approach).

In this paper, we argue that there are important aspects of cultural evolution that do not fit even within a selectional model, and which are better explained and modelled as part of the broader population and evolutionary frames (see also [15]). Specifically, we argue that cultural evolution is best described in terms of a process called cultural attraction [16,17], which is populational and evolutionary, but only selectional under certain circumstances. If we are correct, this has several important consequences for the modelling of cultural evolution, the most important of which is that it implies that in order to model cultural evolution, we must not simply adjust existing replicative or selectional models to fit the cultural case, but we should rather generalize them. Just as replicator-based selection can be seen as one type of Darwinian selection, Darwinian selection can be seen as one of several different types of attraction. Selection, we will argue, is not an alternative to attraction, but rather a special case of it.

Thus, once we have made the argument that cultural evolution proceeds by way of attraction (§§2 and 3), we shall, by way of illustration, introduce a simple formal tool, evolutionary causal matrices (ECMs), that highlights basic properties that might make models of cultural evolution focused on attraction a source of novel insights (§4). In introducing ECMs, our goal is not to present such models in a well-developed form, but more modestly to suggest one way in which it might be possible to do population-level thinking without commitment to a selectional framework. In sum, our objectives here are, first, to illustrate how, at a microscopic level, cultural evolution proceeds by way of attraction, of which selection is a special case; and, second, to sketch one possible way in which it might be modelled in future research.

## 2. Culture as an epidemiological phenomenon

A specific aspect of culture relevant not just to describing it but also to explaining several of its features is its well-recognized ‘epidemiological’ character [3,18]. A population of cultural items inhabits another population, the animal population within which the items propagate. The culture-hosting population is itself an evolving biological population. Just as pathogens and their hosts may co-evolve, so may cultural items and their host. This is especially the case for humans, who have the most developed and enduring cultures and whose biological fitness is closely dependent on their cultural abilities. One of the most important developments in the recent study of cultural evolution has been that of ideas and models of gene–culture coevolution that help flesh out an epidemiological perspective on culture [8].

Comparison with different types of epidemiological phenomena goes together with different views of how culture in general propagates. For example, comparison of cultural transmission with the transmission of infectious diseases suggests that cultural items replicate or reproduce themselves, and this raises the question of how they do so. The mechanism typically invoked is imitation: an individual observing others acquires their behaviour by copying it [2,3,8]. By contrast, comparisons with non-infectious diseases (e.g. psychiatric diseases that can be ‘contagious’ without a pathogenic agent) suggests that the programme for a given type of cultural behaviour may, at least in part, already be latent in potential hosts. In such cases, the behaviour of others (or modifications of the environment caused by others' behaviour) may play the role not of a model but of a trigger, as it does for instance in the case of contagious laughter, or it may play the role both of a model and of a trigger, as it does in the case of the propagation of addictions. These are cases of ‘re-production’ or ‘recurrence’, in the sense of producing one more token of a type, rather than of ‘reproduction’ in the usual sense of copying. Given the diversity of cultural phenomena, comparisons with a variety of both infectious and non-infectious conditions may actually be relevant. In any case, what the comparison with epidemiology suggests is that there may well be a variety of mechanisms and patterns of cultural propagation, and that many of them may not have any close biological analogue.

A simple illustration of this contrast is provided by the case of words, which are cultural items *par excellence*. In acquiring a new word, say ‘dog’, children have to acquire both the sound and the meaning of the word. The actual process of word–sound acquisition is a complex and specialized one, but to say that children learning the word ‘dog’ hear and imitate the sound [d
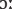
g] is a defensible simplification. On the other hand, describing the acquisition of the meaning of a word as a case of imitation makes little sense. Meanings cannot be observed and imitated; they have to be inferentially reconstructed. The child, for instance, might be able to infer on the basis of contextual evidence and expectations of relevance that the speaker who just said ‘what a nice dog!’ is referring to the terrier they are both looking at. Her task then is to generalize in just the right way the meaning of the word ‘dog’ to all and only dogs (i.e. not also to cats; and not only to terriers), that is, to reconstruct a meaning on the basis of limited evidence and of background knowledge. While the propagation of word sound may be seen as based on copying, that of word meaning cannot: it is re-productive, in the sense that it necessarily involves the triggering of constructive processes.

More generally, the comparison with epidemiology suggests that different cultural items, just as different diseases, may propagate in diverse ways, exploiting whenever possible all the dispositions and susceptibilities of the host population. However, a pervasive idealization in the study of cultural evolution has been that culture is transmitted only or largely through imitation-based copying. No doubt this occurs, but just as it would be very surprising to find an animal population with only viral diseases, it would be very surprising to discover that human culture is made up entirely of items that propagate through imitation, when humans have so many other ways to share information that might also lead to its population-scale propagation. The mechanisms of cultural propagation are instead many and varied, and often involve re-production, or recurrence, rather than just reproduction. This fact, and the epidemiological character of cultural propagation more generally, has, we shall now argue, important consequences for how we understand the dynamics of cultural evolution.

## 3. Preservative and constructive aspects of cultural transmission

Darwin himself knew nothing of the mechanisms of biological inheritance, but he, like everybody else, knew that, generation after generation, animals and plants have descendants of the same species and variety. In other terms, he knew that there had to exist a robust mechanism of biological inheritance.

The case of cultural evolution appears to be similar in several important respects. In particular, there seem to be relatively stable representations, practices and artefacts that are distributed across generations throughout a social group. This stability might seem to be sufficient evidence of the heritability of cultural items. In other words, the very existence of enduring culture seems to suggest that the micro-processes of cultural propagation, whatever they happen to be, are faithful enough to secure a level of heritability that is sufficiently high for selection to operate. Indeed, it may seem that the reality of cultural inheritance is as blatant as the reality of biological inheritance was for Darwin, and relatedly that the actual mechanisms of inheritance are still as mysterious to us as the mechanisms of biological inheritance were to Darwin [7,19,20].

There are two serious problems with this argument. The first is that, whereas the mechanisms of biological inheritance operate at a molecular level, the study of which was not possible at the time of Darwin, the mechanisms of cultural transmission are, to an important extent, accessible to ordinary observation. (It could hardly be otherwise, since cultural transmission itself relies in large part on ordinary individual capacities of observation.) Most of cultural propagation—learning, teaching, sharing of attitudes and values, and so on—takes place through the production and perception of perceptible stimuli; in other words, through the ordinary channels of information transmission, and in particular through imitation and communication, two types of mechanisms of which every ordinary person has a working knowledge. Moreover, imitation, communication and also memory, without which cultural information would not survive to propagate, have been studied in depth by neuroscientists, psychologists, linguists, anthropologists and sociologists. So our knowledge of these mechanisms starts with common sense but goes to some serious scientific depth. Scientific studies have also shown that there are unconscious forms of imitation in human and other animals that play an important role in coordination, in social bonding and in the propagation of some cultural traits, such as linguistic accent and culture-specific bodily postures [21]. All this makes it possible to state with confidence that cultural propagation operates not through one, but through many basic mechanisms of cognition (in particular memory) and transmission (in particular various forms of imitation, communication and teaching). Indeed, the acquisition of cultural items may be one of the proper functions of some of these mechanisms [22].

There is a second and worse problem with the above argument. While some cultural items may indeed be propagated by imitation and other forms of copying, it is clear that a large number are not. In particular, many are also (re-)constructed. For example, a student taking notes in a lecture does not simply copy any spelling error that the lecturer happens to write down, but will in fact, in her own notes, correct the error and in doing so re-construct the correct spelling. As such, cultural propagation is partly preservative, but also partly (re-)constructive, to different degrees in each particular case. As such, it is not only a matter of inheritance, as is generally the case for biology, but also of reconstruction. Whichever of these is more important in any given case is an empirical question, but either way, the direct analogy with biological evolution is considerably weakened by this fact.

Furthermore, and quite generally, imitation is not a goal in itself but a means towards some other goal, cognitive or practical. After all, not everything we observe, understand or remember is useful. On the contrary, it is often efficient to be selective and ignore some of it. The level of fidelity in preservation aimed at is that level which is appropriate to the pursuit of this goal. Sometimes, this will be high: if a child wants to tie her shoes on her own, she is more likely to succeed by faithfully imitating a model rather than by trying to construct an appropriate knot on her own. At other times it will be low: if an experienced cook wants to adopt a new recipe, he is likely to adapt it from the start to his own taste rather than aim at strict imitation. Both the child and the cook participate in cultural propagation even though this is not in and of itself their goal, and even though only the child aims at copying. In other words, the outputs of individual memory, imitation and communication processes are not copies but modifications of the inputs. This is due in part to the imperfection of these mechanisms: some information is just lost in the process of transmission. But more importantly, the mechanisms involved in cultural transmission (with rare exceptions such as rote learning) have, in various degrees and forms, both preservative and constructive functions. This is quite unlike the biological case, in which the proper function of the copying mechanism (replication) is preservative alone. The good news is that the constructive character of these mechanisms of cultural propagation, rather than being a problem, is in fact, as we shall now explain, a key element in explaining cultural stability [23]. As such, it is not something that we should always try to idealize away from, as models in the selectional frame necessarily do. Rather, we should look to incorporate it into our models of cultural evolution.

If all transmission processes were just preservative, an occasional error of replication (akin to a mutation), such as the lecturer's spelling error, would be preserved in further errorless replications: it would become the model. If such copying errors were frequent, heritability might be too low for selection to be effective, or for anything much to stabilize at all. In such circumstances, there are two factors that might nevertheless maintain cultural stability. One is a transmission factor: people may either copy several models and average across their differences thus eliminating idiosyncratic variations, or they may all preferentially copy the same models. The effects of either of these procedures may to some extent neutralize the cumulative effects of any copying errors [8,24,25]. The second factor is that the constructive processes we discussed above may tend to transform different inputs in similar ways (rather than randomly), and in doing so cause the outputs to tendentially converge upon particular types, called attractors. This tendency is called cultural attraction.

Here is an example. The region of the continuum of colours referred to by a given colour term, say ‘red’, does not have clear boundaries, but it has, for every user, a focal point which is seen as prototypical red. In learning the meaning of ‘red’, a child is not taking the first sample she hears described as ‘red’ as prototypical red, nor is she averaging over all the samples that she hears described as ‘red’ in her learning period. Rather, her colour perception system influences her interpretation of the word. She may depart from the samples of ‘red’ she is provided with, in the direction of what is a more salient identification given her perceptual dispositions. Because these perceptual dispositions for red and other basic colours are very similar across individuals, they stabilize common meanings for basic colour terms in any given language, and terms with the same colour spectrum across many languages [26–28]. By contrast, the meanings of non-basic colour terms, for instance ‘crimson’ or ‘indigo’, are not similarly stabilized by cognitive biases, and they are hence often interpreted with a high degree of idiosyncrasy and are more language-specific. These non-basic colour terms are instead sometimes stabilized by the presence in the environment of some culturally salient items that exemplifies the colour, for instance indigo-dyed cloth, and in some cases they are not especially stable at all. Further examples are not difficult to find.

Many constructive biases are shared in a population. In the example above, the source of the bias was psycho-physical (the colour perception system). Another common biological source of biases is human psychology. A third is specific historical or environmental factors that cause individuals to interpret inputs in locally converging ways. If, for instance, one cultural trait is already present in a population, that can favour or hinder the propagation of other traits. Shamanism and the consumption of hallucinogenic substances, for instance, even though they can exist independently of one another, are two mutually supportive cultural traits: shamanism provides an institutional framework and a positive interpretation of hallucinogenic experiences thus reinforcing the practice. The hallucinations themselves provide evidence of shamanistic powers thus reinforcing the institution. Another kind of example is provided by the adaptation of techniques to a local environment. Fishermen, for instance, use hooks adapted to catching the fish that are locally available. While trial and error no doubt plays a role in the evolution of hooks, constructive mental processes that imaginatively anticipate the effects of hook design and size and evaluate them are no less important. Such ‘guided variations’, as the phenomenon is recognized and described [2], are just one important type of constructive processes. When constructive biases are shared throughout a population they may, whatever the source of the bias, permit some types to reach a cultural level of distribution and stability, as in each of the examples above, and they may do so despite the low fidelity of preservative micro-processes.

Both transmission factors and constructive factors provide an explanation of macro cultural stability in spite of low micro fidelity. On the one hand, transmission factors, such as averaging across models, or preferential copying of certain favoured models, may neutralize low fidelity; they do so without favouring one type of content over another. On the other hand, constructive factors such as those discussed above may secure stability not by neutralizing low fidelity but instead by introducing directionality and convergence in transformations and thereby counteracting randomness. Unlike transmission factors, constructive factors make some specific contents more likely to evolve and stabilize than others. The relative importance of these two types of factors in any given case is an open empirical question but these two explanations of cultural stability have quite different consequences for the modelling of cultural evolution. As has been previously shown, transmission factors can be integrated in selectional models [2,3,8]. Constructive factors, on the other hand, call for a different type of populational modelling, one that explains the dynamics of cultural evolution not in terms of reproductive success but in terms attraction. In §4, we suggest one way in which such models might be developed.

## 4. Evolutionary causal matrices

Our goal in this section is to sketch one way in which the sort of approach we have outlined above might be modelled. We want in particular to show how selection can be viewed as a special case of attraction. To do this, we present a simple formal device that we use here as a tool to sharpen ideas and make them more intuitive. It can be seen as either what Dennett calls an ‘intuition pump’ [29], or as a rudimentary sketch of an actual model. We hope that it may be suggestive of a genuine formal treatment in the future, but this is not our present goal, which is instead to simply illustrate and make more explicit the sort of project we have in mind.

The main idea is that of an ECM [30]. Consider a population P of items of various types that evolves over time and that each may have an impact on the evolution of the others. This will be in particular the case for sub-types that are variants of a more general type (e.g. pouring the milk in the cup before or after the tea; saying ‘I have proved’ or ‘I have proven’), and types that are complementary of one another (e.g. drinking tea and using a tea cup; praying and wearing a religious symbol). When we say that P evolves, we mean two things: (i) that the frequency of types in P changes over time and (ii) that the frequency of types at a given time step is a function of their frequencies at earlier time steps. (We make the idealization that time can be partitioned in discrete steps.) For simplicity, here we only consider the case where frequencies at time *t* are just a function of frequencies at time *t* − 1 (rather than being also a function of frequencies at earlier time steps).

In order to model the process of cultural attraction, we must represent the possibility that every item of every type at *t* may have some causal effect or, as we will call it, impact, on the frequency of items of every type at *t* + 1 (where frequency is measured in absolute numbers, rather than relatively). In the general case, the occurrence of every item of every type at *t* has some probabilistic impact on the frequency of items of every type at *t* + 1. To represent this general case, we use an ECM which is an *N_T_* × *N_T_* square matrix where *N_T_* is the number of types in P and where the coefficient *I_XY_* in each cell represents the average impact that each item of type *X* at *t* has on the frequency of type *Y* at *t* + 1. Table 1 represents the ECM of a population with three types, *A*, *B* and *C.*

**Table 1.** An ECM for three types, *A*, *B* and *C. I_AB_* represents the average impact of one item of type *A* on the frequency of items of type *B*.

	*A*(*t* + *1*)	*B*(*t* + *1*)	*C*(*t* + *1*)
*A*(*t*)	*I_AA_*	*I_AB_*	*I_AC_*
*B*(*t*)	*I_BA_*	*I_BB_*	*I_BC_*
*C*(*t*)	*I_CA_*	*I_CB_*	*I_CC_*

The impact of *X*s on frequencies of all the types in the population P is represented by the *X* row. The impact that items of every type in P have on the frequency of *Y* is represented by the *Y* column. As such, the frequency of *Y*s at *t* + 1 is determined by the impact that all the different types have on the frequency of *Y*s, and on the frequency of each of those different types at *t*. (In this sense, there is a simple form of frequency-dependence built into this model.) When all of these values are known, the relative frequency of *Y*s at *t* + 1, *F_Y_*(*t* + 1), can be straightforwardly computed:4.1
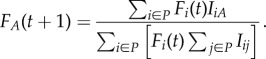


If we label the function in equation (4.1) as g(*f_A_*(*t*)), then over time each type A will tend towards a frequency given by 
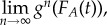
 i.e. the limit, as *n* tends to infinity, of iteratively applying *g* to itself *n* times. We can then characterize an attractor as any type whose relative frequency tends to increase over time. These equations make it clear that: (i) whether or not a given type is an attractor depends on its relative success at benefitting not only from the impact and frequency at the previous time step of items of the same time, but in fact of the impact and frequency at the previous time step of all the members of the population, whatever their type; and (ii) the frequency of one type depends on the impacts of other types *on others*, and not just of others on the focal type. (These facts will of course lead to further frequency-dependence effects, which we do not explore here.) Consequently, the same type may be an attractor within one population, but not in another.

We now provide a number of simple, miscellaneous illustrative examples of ECMs, and the modelling possibilities they afford. To the extent that we are only interested in the evolution of the relative frequency of the types, we could use arbitrary absolute numbers for these *I_XY_* coefficients as long as their values relative to one another are appropriate. For the sake of clarity, however, we will use values that can be meaningfully interpreted: here, *I_XY_* is the average additional number of *Y* at *t* + 1 that results from the occurrence of each *X* at *t*, ignoring constraints on the size of the overall population P. In such conditions, *I_XY_* = 1 means that each *X* at *t* adds one *Y* at *t* + 1; *I_XY_* = −1 means that each *X* at *t* subtracts one *Y* at *t* + 1 (with, for obvious reasons, any negative frequency value being normalized to zero); and so on.

ECMs can be used in a variety of different ways. In particular, the *N_T_* different types in P could be either mental or public (i.e. beliefs about how to tie a knot; or actual knots), and as such, ECMs could be used to track the evolution of either type of representation. Mental and public representations can also be combined in the same matrix. We give examples of all of these possibilities in what follows. The ECM format also makes it easy to single out and describe interesting special cases, of which we will mention a few.

We begin with a concrete and simplified example. Consider the case of the Latin word ‘data’ being borrowed in an English-speaking community and pronounced dātә (‘dar-ta’) or dätә (‘day-ta’), with the first syllable being pronounced initially as the Latin-sounding dā but, more and more frequently, as the English-sounding dä. Imagine a community where the causal relationships between these two pronunciations could be represented as in table 2. In this case, both pronunciations have exactly the same impact on the frequency of the same type at *t* + 1. However the positive impact of dā on dä is greater than that of dä on dā (4 versus 0.1), because dä fits better with the existing English phonological system than does dā. As a result, people who acquire the word mostly from people who pronounce it dātә are much more likely to themselves pronounce it dätә than conversely. With an initial frequency of dä = 0.001, one can simulate the evolution of the system. After 40 time steps, the system stabilizes with 14% of utterances of the word ‘data’ being pronounced dātә, and 86% dätә.

**Table 2.** ECM of the two pronunciations of ‘data’.

	dā(*t* + 1)	dä(*t* + 1)
dā(*t*)	5	4
dä(*t*)	0.1	5

We now illustrate how ECMs relate to existing models, beginning with the basic case of Darwinian selection. Consider the I*_XX_* cells on the upper left/lower right diagonal in an ECM. They represent the impact that items of type *X* at *t* have on the frequency of the same type *X* at *t* + 1 (a causal effect we may call homo-impact, distinguishing it from relationships of hetero-impact among items of different types). If practically all the impacts on items in P are homo-impacts, as in table 3, then the values in the diagonal can be interpreted as reproduction rate, and determine the evolution of the system in the standard selectional way. Negligible values in the cells that are not on the diagonal can be interpreted as a mutation rate. Note that in such cases equation (4.1) reduces to the discrete replicator dynamics, 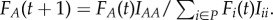

**Table 3.** The reproduction case. As items are reproduced from one time step to the other (except for the rare mutations) the highest value in the diagonal predicts the outcome (here *B* will invade the population and *A* will remain in small proportion).

	*A*(*t* + 1)	*B*(*t* + 1)
*A*(*t*)	4	0.001
*B*(*t*)	0.001	5

Quite generally, previous models of cultural evolution have made the simplifying assumption that cultural items reproduce with rates of mutation that, although higher than those found in gene replication, are still low enough, given selection pressures, to secure the dynamics of Darwinian selection. We have argued that this assumption is not based on empirical evidence that cultural items in general propagate through copying, as opposed to other forms of transmission that involve a mix of preservative and constructive processes. It has nevertheless been a profitable idealization, given the subsequent successes of those models. Modelling within this idealization does not need and would likely not benefit from a matrix format of the type we are suggesting. The point of introducing this case here is rather to show that Darwinian selection is a special case of this more general class of model, and not an alternative to them. We shall return to this point in the summary.

We now consider various matrices of different formats that are relevant to the study of cultural evolution in different ways. One such case is ECMs where cells on the upper left/lower right diagonal have a zero value. That is, homo-impact is nil, and the evolution of the type depends exclusively on hetero-impact. This pattern is common if one considers with sufficiently high resolution the causal chains of cultural propagation. Consider an orally transmitted folktale. The public telling of the tale contributes to the formation in the listeners of mental representations of the tale, and these mental representations contribute to the production of further public telling by listeners turned tellers. Some authors have suggested that the mental versions are the true memes and as such the cultural counterpart of genes, while the public tellings are mere phenotypes, but the analogy with the gene is weak: there is no ‘memic’ counterpart of a germline linking the mental representations in different individuals. The causal chains instead involve an alternation of mental and public events with equally potent causal roles (a distant biological analogue may be found in the case of RNA retroviruses that propagate through reverse transcription into DNA). Table 4 represents in a much simplified way the causal relations between mental representations (MR) and public telling (PT) of a folktale.

**Table 4.** The folktale case. Here, each person tells the story to an average of six listeners each, and each of those listeners has to hear the tale five times on average before they remember it properly.

	MR(*t* + 1)	PT(*t* + 1)
MR(*t*)	0	6
PT(*t*)	0.2	0

Note that the folktale case so described can also be accommodated within a selectional framework by approaching it in a more coarse-grained way. For this, one should pick, even if at the cost of some arbitrariness, either the mental or the public versions of the tale as the ‘real’ cultural items and consider the alternation in the propagation process of mental and public version as an aspect of the mechanism of propagation that can be ignored in the evolutionary model. To go this way, one has to idealize away the facts that (i) tellings of a tale are not identical to one another, (ii) mental versions are constructive syntheses of the interpretation of several tellings rather than mere registrations and (iii) tellings are not public production of an internally memorized text but construction of a new public version on the basis of memorized information that is not in text form. For the reasons already argued in this paper, we think that interesting and important aspects not just of the mechanics of the propagation of folktales but also of their evolutionary dynamics are missed when these facts are idealized away.

Two other interesting cases are provided by pairs of items that have exactly the same impact on themselves and on each other, as in tables 5 and 6.

**Table 5.** Conformity bias. If one or the other item prevails at one time (because of stochasticity for instance) it will become more and more frequent due to a negative influence on the other type.

	*A*(*t* + 1)	*B*(*t* + 1)
*A*(*t*)	1	−0.1
*B*(*t*)	−0.1	1

**Table 6.** The two-party system: whatever the initial frequencies, the system tends towards an equal frequency of the two types because of the positive impact that each type has on the other.

	*C*(*t* + 1)	*D*(*t* + 1)
*C*(*t*)	1	0.1
*D*(*t*)	0.1	1

In the case of table 5, in spite of equality of impact on *A* and *B*, the type that happens to be more frequent is likely to drive out the other, as its cumulative negative impact on the other type will be greater than the cumulative negative impact it suffers from, and more so at every step in time. This distribution of causal powers yields an effect similar to the conformity bias well described by Boyd & Richerson [2]. In the case of table 6, even if the frequency of *C* and *D* are initially quite different, the equilibrium point is one of equal frequency between the types, since, through its cumulative impact, the more frequent type benefits more than the less frequent type rather than the other way around. An example of this is provided by the two-party political system where, say, party *C* not only attracts the votes of its own followers but also causes citizens to vote for the other party D not so much because they are positively influenced by D but because they want to vote against *C*.

## 5. Summary

How deep is the analogy between biological and cultural evolution? Memetics assumes that it is deep indeed; that the main relevant details of the biological case have direct equivalents in the cultural case, such that there is, for example, a cultural phenotype, which achieves a certain level of (inclusive) fitness, which will in turn determine the phenotype's relative success in the population. Selectionist approaches loosen the analogy somewhat, moving from a replicative frame to the more general selectional frame (figure 1). We have argued that the analogy should be loosened further: cultural evolution is broadly Darwinian, in the sense that it is a population-level evolutionary phenomenon, but there is no empirical reason to think that it sits entirely or even in general within the selectional frame.

Another important disanalogy between biological and cultural evolution is the mechanisms by which traits propagate through a population. In biology, the mechanisms of transmission are in general only preservative. In the cultural case, however, the mechanisms of transmission are many and varied, and include both preservative and constructive sub-mechanisms. Constructive sub-mechanisms are common and, because they are often shared within a population, they often transform cultural traits in systematic ways, such that they converge upon particular types, which we call attractors. The process by which they do this is called cultural attraction. This provides an explanation of cultural stability that is more general than explanations based on preferential selection (which are incorporated as a special case).

Both attractors and the process of attraction are statistical notions. They do not denote a type of causal process or the outcome of a specific such process, and as such they do not provide explanations of cultural phenomena. Rather, they provide relevant descriptions of what is to be explained. Attraction should instead be explained in terms of factors of attraction. Factors of attraction in an epidemiological population will generally be partitionable into two classes: relevant properties of the individual members of the host population (such as the psychological and biological susceptibilities of humans); and relevant properties of the environment of these individuals, including the demographic properties of the host population itself. For instance, the evolved phonological capacities of humans and the acquired phonological competence in one's native tongue are examples of psychological factors of attraction in the propagation of word sounds; the availability in the natural environment of hallucigenic substances and the practice of using them in the population are ecological factors in the propagation of shamanism.

Reproductive success is a special aspect of attraction, rather than an alternative to it. Specifically, a selected trait is an attractor that owes its higher frequency mostly to homo-impact, i.e. reproductive success. As such, the key questions to ask about selection are not about its importance relative to attraction, as if attraction and selection were alternative explanations, but rather what part of attraction, if any, is due to selection. The answer will be different for different traits and situations. For instance—drawing on examples discussed—reproductive success is generally likely to be a more important factor of attraction in the proliferation of word sounds than in that of word meaning. Among word sounds, learning biases based on language-specific phonological regularities are likely to be a more important factor of attraction and reproductive success to be a less important factor for recently borrowed words (such as ‘data’) than for native words.

Darwinian selection leads to the maximization of inclusive fitness, and this explains the appearance of design in the natural world [31]. Is there an analogous result for cultural attraction? As selection is a special case of attraction, design is possible and in some cases explicable in standard Darwinian terms. Having said that, such explanations will not apply generally, and may not even apply commonly. However, design can emerge in cultural evolution in another way, not as the direct result of selection, but instead because the epidemiological character of cultural evolution means that cognitive (and other biological) factors of attraction may cause cultural items to tendentially evolve towards greater design. These biological factors of attraction may themselves be adaptations, that is, outputs of natural selection. Their presence biases cultural transmission towards representations, practices or artefacts that have an optimal design for the biologically evolved adaptation that makes use of them. In other words, attraction can also result in design as an indirect (proximate) effect of the natural selection of factors of attraction. One experimental illustration of this is the cultural evolution of song types in Zebra finches [32], and one real world example is the gaze of sitters in portraiture: humans are particularly sensitive to direct eye gaze, and in cultures where a portrait sitters' gaze direction is left free to vary, the culturally preferred form tends, over time, to move in the direction of direct eye gaze, and away from averted gaze [33]. A general, formal statement of what cultural attraction leads to does not presently exist, and we see the development of such a statement as a major goal for future modelling work.

More generally, the arguments we have developed here collectively raise the question of how cultural evolution should be modelled to take account of attraction and attractors. Our sketch of ECMs (§4) is not meant to be an adequate formal tool for such modelling. Our goal was instead to highlight the sort of properties that a general model of cultural evolution should have if it is to represent the populational effects of constructive processes in cultural propagation. Our hope is that future research will include the development of sophisticated models of this type.

## Funding statement

T.C.S-P. gratefully acknowledges financial support from the ESRC; N.C. from the Agence Nationale de la Recherche.
